# Predicting Risk of Suicide Attempt Using History of Physical Illnesses From Electronic Medical Records

**DOI:** 10.2196/mental.5475

**Published:** 2016-07-11

**Authors:** Chandan Karmakar, Wei Luo, Truyen Tran, Michael Berk, Svetha Venkatesh

**Affiliations:** ^1^Centre for Pattern Recognition and Data AnalyticsDeakin UniversityGeelongAustralia; ^2^Barwon HealthGeelongAustralia

**Keywords:** suicide risk, electronic medical record, history of physical illnesses, ICD-10 codes, suicide risk prediction model

## Abstract

**Background:**

Although physical illnesses, routinely documented in electronic medical records (EMR), have been found to be a contributing factor to suicides, no automated systems use this information to predict suicide risk.

**Objective:**

The aim of this study is to quantify the impact of physical illnesses on suicide risk, and develop a predictive model that captures this relationship using EMR data.

**Methods:**

We used history of physical illnesses (except chapter V: Mental and behavioral disorders) from EMR data over different time-periods to build a lookup table that contains the probability of suicide risk for each chapter of the International Statistical Classification of Diseases and Related Health Problems, 10th Revision (ICD-10) codes. The lookup table was then used to predict the probability of suicide risk for any new assessment. Based on the different lengths of history of physical illnesses, we developed six different models to predict suicide risk. We tested the performance of developed models to predict 90-day risk using historical data over differing time-periods ranging from 3 to 48 months. A total of 16,858 assessments from 7399 mental health patients with at least one risk assessment was used for the validation of the developed model. The performance was measured using area under the receiver operating characteristic curve (AUC).

**Results:**

The best predictive results were derived (AUC=0.71) using combined data across all time-periods, which significantly outperformed the clinical baseline derived from routine risk assessment (AUC=0.56). The proposed approach thus shows potential to be incorporated in the broader risk assessment processes used by clinicians.

**Conclusions:**

This study provides a novel approach to exploit the history of physical illnesses extracted from EMR (ICD-10 codes without chapter V-mental and behavioral disorders) to predict suicide risk, and this model outperforms existing clinical assessments of suicide risk.

## Introduction

Suicide is a prominent public health concern. All over the world each year, 2% of the population contemplate suicide [1]. In 2013, an average of 6.9 suicide deaths was recorded in Australia each day. It is estimated that by 2020 suicide will become the 10^th^ most common cause of death in the world [2]. Therefore, suicide prevention is important and is an active research field. Because general practitioners are usually the first port of call for mental health problems, the suicide prevention process should be integrated within both hospital treatment and general medical practice [3].

In the last decades, large epidemiological studies have identified the number of previous suicide attempts, lethality of previous attempts, psychiatric disorders, and social isolation as potential risk factors for suicide [4-9]. Besides identifying independent risk or protective factors, these epidemiologic studies also quantified the strength of their relative contribution. Despite the effort to combine these risk factors into risk scores and algorithms to predict suicide risk [10-12], the predictions often have sensitivity and specificity that are too poor to be clinically useful [13,14]. The failure of these approaches may be attributed to the complex nature of suicidal behavior, which consists of an evolving and multifactorial constellation of components that act together but vary from one individual to another. On the other hand, clinical assessment of suicide risk is primarily done based on the response of the patient, where current suicidal ideation and known risks are integrated. Although suicidality is a prominent risk factor for suicide attempts and completion, only approximately 30% of patients attempting suicide disclose their suicidal ideation [15-17] and the vast majority of individuals who express suicidal ideation never attempt suicide [18-20].

To improve the clinical assessment or predictive value of suicide risk, researchers have started to look at the broader source of available information, such as electronic medical records (EMR) [21] and clinical notes [22]. Recent papers showed that EMR can be used to predict various medical conditions including chronic obstructive pulmonary disease in asthma patients [23], genetic risk for type 2 diabetes [24], myocardial infarction [25], 5-years life expectancy of elderly population [26], and 30-day life expectancy of cancer hospitalized cancer patients [27]. In our previous work [21], we have developed a statistical risk stratification model to predict attempt of suicide risk based on EMR data and the model performance was found to be better than clinical predictions based on an 18-point risk assessment instrument. However, this model was complex and does not generalize to facilitate limited routine data collection. Moreover, because EMR contains a wide variety of information, there is a strong possibility that combinations of them can be infinite. Poulin et al [22] have developed linguistics-driven prediction models to estimate the risk of suicide using unstructured clinical notes taken from a national sample of US Veterans Administration medical records. From the clinical notes, they generated datasets of single keywords and multiword phrases, and constructed prediction models using a machine-learning algorithm based on a genetic programming framework. Although their result showed an accuracy of 65% or more, it was based on a small veteran population and the method was too complex to derive any symptomatic link with the suicide risk factors.

Recently, Qin et al [28] analyzed the relationship between suicidal death and physical illness, which was the first detailed analysis where physical illness was categorized based on the International Statistical Classification of Diseases and Related Health Problems, 10th Revision (ICD-10) chapters. The results of the study showed that the frequency of hospitalization elevates the risk of suicide deaths and this relationship was significant. However, the study is different from this study in terms of population groups and length of history of physical illness used for analyzing the cohort.

In this study, using machine learning to analyze EMR, we aim to find the effect of physical illnesses for the at-risk population, considering patients who have received at least one suicide risk assessment but who did not attempt suicide. We hypothesize that the relationship between physical illnesses alone and suicide risk can be exploited for quantitative assessment of suicidal risk using ICD-10 codes. This means that we do not use the “Mental and behavioural disorders” (Chapter V, ICD codes), relying purely on the physical illnesses. We developed a novel predictive model to obtain a suicide risk score using the history of physical illnesses derived from ICD-10 codes. Finally, we compared the performance of the physical-illness-based risk score with corresponding baseline clinical assessment.

## Methods

### Data

#### Data Description

The data was collected retrospectively from the EMRs, coded using ICD-10-AM, within Barwon Health, Australia [21]. This is a regional hospital serving an area of 350,000 residents. The data consisted of 7399 mental health patients who were 10-years or older and were underwent assessment for suicide risk between April 2009 and March 2012. There were 16,858 assessments, each of which was considered as an observational case, from which suicide risk could be predicted. In the follow-up period of 90 days after an assessment, the ground truth of suicide risk levels were determined through ICD-10 codes occurring during the period. In this study, we have divided the complete population into Control and Risk groups. The Control group consists of assessments of patients who never attempt suicide. Thus, the Risk group consists of assessments of patients who commit at least one suicide attempt.

Ethics approval was obtained from the Hospital and Research Ethics Committee at Barwon Health (number 12/83). Deakin University has reciprocal ethics authorization with Barwon Health. Although all patients has given written informed consent, patient information was anonymized and de-identified prior to analysis.

#### Clinical Risk Scoring

Suicide risk assessments were routinely performed by clinicians using an instrument developed internally. The instrument has been in use for 15 years. The checklist has the following 18 items: suicidal ideation, suicide plan, access to means, prior attempts, anger/hostility/impulsivity, current level of depression, anxiety, disorientation/disorganization, hopelessness, identifiable stressors, substance abuse, psychosis, medical status, withdrawal from others, expressed communication, psychiatric service history, coping strategies, and supportive others (connectedness).

Based on the ratings (from 1-3) for these 18 items, an overall rating of suicide risk (*RiskScore_clinical_*) is determined on a scale from 0 (lowest) to 4 (highest). For the purpose of this study, the overall rating was used as the baseline for comparison. A total of 15,513 assessments had *RiskScore_clinical_*˃ 0, which is approximately 92.02% (15513/16858) of total assessments used in this study.

#### Selection of ICD-10 Chapters and Calculating Frequency of Physical Illnesses

ICD-10 (2015 version) has 22 chapters to code all diseases recorded in EMR. The following shows the exclusion and inclusion chapter that were used.

##### Exclusion

We removed all codes from chapter V, that is all codes related to “Mental and behavioral disorders.” We removed chapters XVI (Certain conditions originating in the perinatal period) and XVII (congenital malformations, deformations, and chromosomal abnormalities) codes altogether as these were absent in the studied population.

##### Inclusion

We merged chapters VII (Diseases of the eye and adnexa) and VIII (Diseases of the ear and mastoid process) due to minimal presence of these diagnostic codes. As a result, we finally have 18 chapter headings (ch=1,2,…,18) corresponding to 19 ICD-10 chapters.

##### Computing Frequency of Codes for Each Chapter

**Figure 1:**
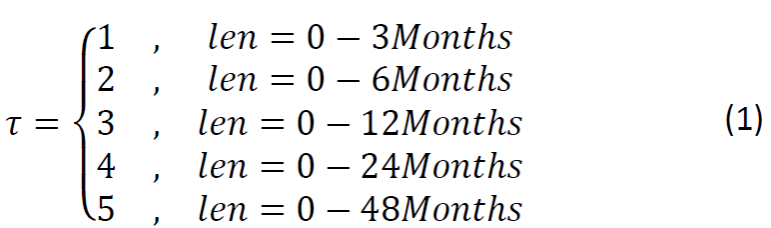
Equation 1.

**Figure 2:**
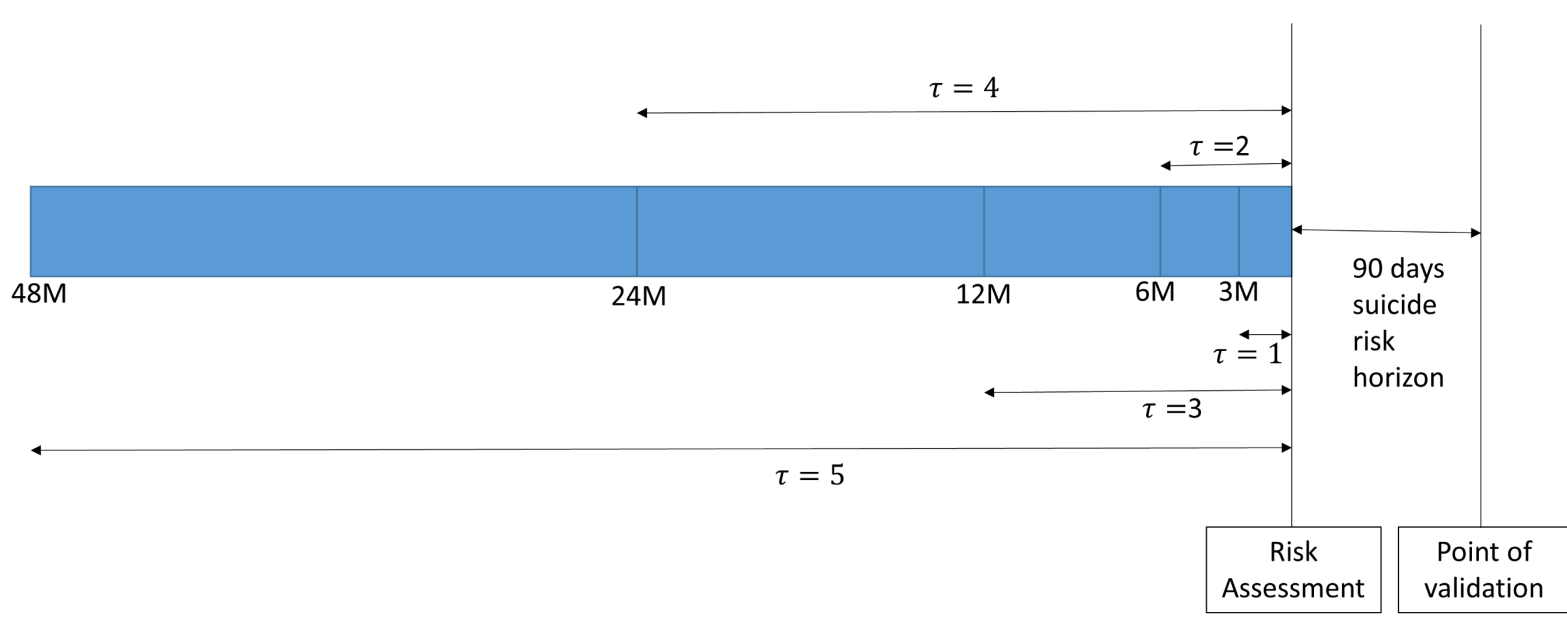
Time periods used to extract the history of physical illnesses from EMR and the suicide risk prediction horizon.

We first defined the time-period (*len*) over which the patient history EMR was included. Five different time-periods were used (Figure 1 and 2):

For each time-period, the ICD-10 codes for each assessment were aggregated under selected chapters, wherein each aggregated value represents the total number of occurrences of physical illnesses for the corresponding chapter. Therefore, for each assessment *i* we obtained a vector *f*_*i*_ *(τ, ch)*, where *ch=1,2, …,18* and *τ=1,2,…,5*. Assessments with an entry of 0 for all 18 chapter headings, were counted as an assessment with no history of physical illnesses. For all assessments, we constructed the matrix *F*_*i*_ *(τ, ch)*, as:

F_i_(τ, ch)f_i_(τ, ch)]^n^_i_=1   (2)

where, *n* is the total number of assessments.

In this study, we developed six models to predict suicide risk based on different length of history of physical illnesses. Five models used the frequency of physical illnesses for the designated time-period, that is, *F(τ, ch)*, where *τ=1,2,…,5* and the sixth model horizontally concatenated frequency matrices from all five individual time periods, *F=[F*_*i*_ *(τ, ch)]*^*5*^_*τ=1*_.

#### Creating the Suicide Risk Lookup Table

##### Definition

**Figure 3:**
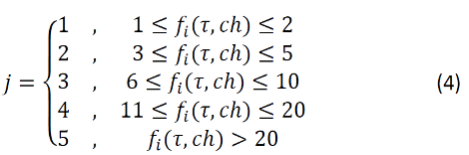
Equation 4.

A probability lookup table *PT* is generated from the history of physical illnesses as:

PT=(pt_t,ch,j_)   (3)

where *τ=1,2,…5* in the index of the time period used to extract history of physical illnesses, *ch=1,2,…,18* is the index of the ICD-chapter and *j=1,2,…,5* is index of the frequency bin. Each element of this table, *PT=(pt*_*t,ch,j*_) is the suicide risk probability of the *ch*^*th*^ chapter defined using historical data from time period *τ* for the *j*^*th*^ frequency bin. To calculate *PT=(pt*_*t,ch,j*_ *)*, we computed the histogram *Hist*_*j*_ *(F*_*i*_ *(τ, ch))*, where *j* is the bin index and defined as in Figure 3:

To separate out the Control and Risk histogram, we introduced the notation *Hist*_*j*_^*Control*^ and *Hist*_*j*_^*Risk*^, which were defined as:

Hist_j_^Control^(τ, ch)=Hist_j_(F_i_(τ,ch))   (5)

where, *i ∈ Control* and *f*_*i*_ *(τ,ch)* ≠0

Hist_j_^Risk^(τ,ch)Hist_j_(F_i_(τ,ch))   (6)

where, *i ∈ Risk* and *f*_*i*_ *(τ,ch)* ≠0

**Figure 4:**
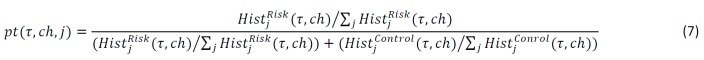
Equation 7.

Finally, the suicide risk probability of *ch*^*th*^ chapter for time period *τ* and bin index *j* was defined by equation 7 in Figure 4.

#### Scoring Suicide Risk of an Assessment

**Figure 5:**
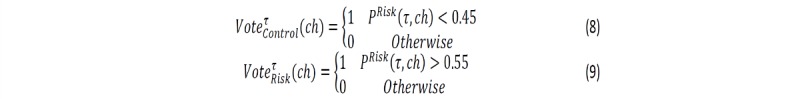
Equations 8 and 9.

**Figure 6:**
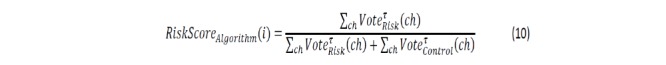
Equation 10.

The suicide risk score (*RiskScore_Algorithm_*) for any assessment was inferred from the suicide risk lookup table *PT*. This was accomplished using following steps:

1. For a new assessment I and historical time-period τ, extract the frequency of physical illness *f*_*i*_ *(τ, ch)* from the EMR data for that assessment.
2. For each chapter *ch*
  - Calculate bin index from *f*_*i*_ *(τ, ch)* using equation 5.
  - Extract *P^Risk^*(τ, ch)=pt_τ,ch,i_ from the lookup table *PT.*
  - Use a Heavyside step function to convert *P*^*Risk*^ *(τ, ch)* into equations 8 and 9 (Figure 5).
3. Calculate suicide risk score *RiskScore_Algorithm_* *(i)* as in equation 10 (Figure 6).

### Performance Evaluation

The performance of ICD-10 code history based suicide risk scores, clinically evaluated scores and their combination were measured using area under the receiver operating characteristic curve (AUC). For the clinical score (*RiskScore_Clinical_*), the performance was evaluated by directly measuring the AUC of the entire assessments without dividing them in training or testing sets.

On the other hand, for *RiskScore_Algorithm_* we used 90% (13,962/15,513) of the assessments to generate reference lookup table that is, training set and the remaining 10% (1551/15,513) of the population was used as a test set to measure AUC. This process was repeated 10 times, where there were 10 different test sets and union of them encompass the original population, and the overall performance was presented by the average AUC that were obtained over those reiterations.

A similar approach (13,962/15,513, 90% training and 1551/15513, 10% test populations with 10 reiterations) was used for evaluating the performance of combination of clinical and ICD-10–based score. Multilinear regression was used to combine two variables. The training data set was used to train the regression model and output was generated using test set and the trained model. Finally, the AUC was computed using the output (*RiskScore_Combined_*) of the model.

## Results

This study included 2072 suicide risk cases and 14,786 control cases, comprising of 1080 male and 992 female suicide risk cases and 7215 male and 7571 female control cases. In the study population, the percentage of suicide risk and control cases over five different time ranges with a history of hospitalization were (1515/2072, 73.12% and 6674/14,786, 45.14%), (1659/2072, 80.07% and 7877/14,786, 53.27%, 1882/2072, 87.93% and 9241/14,786, 60.50%, 1910/2072, 92.18% and 10,794/14,786, 73.00% and 1987/2072, 95.90% and 12,285/14,786, 83.09% over the past 3, 6, 12, 24, and 48 months, respectively (Table 1). This indicates that the number of subjects having physical illness is higher in suicide risk population than the control population irrespective of the time range. In addition, although the percentage of population having no ICD codes decreased with increasing time range in both the control and risk groups, it was reduced to 4.10% (85/2072) in risk group in contrast to 16.91% (2501/14,786) in control group.

**Table 1:** Distribution of physical illnesses according to ICD-10 category (without chapter V-Mental and behavioral disorders) in control and risk groups over five different time ranges.

	τ=1 (0-3 months)				τ=2 (0-6 months)				τ=3 (0-12 months)				τ=4 (0-24 months)				τ=5 (0-48 months)			
	Control		Risk		Control		Risk		Control		Risk		Control		Risk		Control		Risk	
	n	%	n	%	n	%	n	%	n	%	n	%	n	%	n	%	n	%	n	%
**History of ICD codes**																				
No	8112	54.86	557	26.88	6909	46.73	413	19.93	5545	37.50	250	12.07	3992	27.00	162	7.82	2501	16.91	85	4.10
Yes	6674	45.14	1515	73.12	7877	53.27	1659	80.07	9241	62.50	1822	87.93	10794	73.00	1910	92.18	12285	83.09	1987	95.90
**Frequency of ICD codes**																				
0	8112	54.86	557	26.88	6909	46.73	413	19.93	5545	37.50	250	12.07	3992	27.00	162	7.82	2501	16.91	85	4.10
1-2	2914	19.71	500	24.13	2925	19.78	430	20.75	2881	19.48	365	17.62	2733	18.48	238	11.49	2517	17.02	179	8.64
3-5	1641	11.1	319	15.40	1870	12.65	305	14.72	2044	13.82	277	13.37	2213	14.97	254	12.26	2379	16.09	216	10.42
6-10	1317	8.91	356	17.18	1666	11.27	315	15.20	1999	13.52	332	16.02	2286	15.46	336	16.22	2532	17.12	267	12.89
11-20	657	4.44	247	11.92	1028	6.95	366	17.66	1499	10.14	345	16.65	2089	14.13	386	18.63	2338	15.81	383	18.48
>20	145	0.98	93	4.49	388	2.62	243	11.73	818	5.53	503	24.28	1473	9.96	696	33.59	2519	17.04	942	45.46

**Figure 7:**
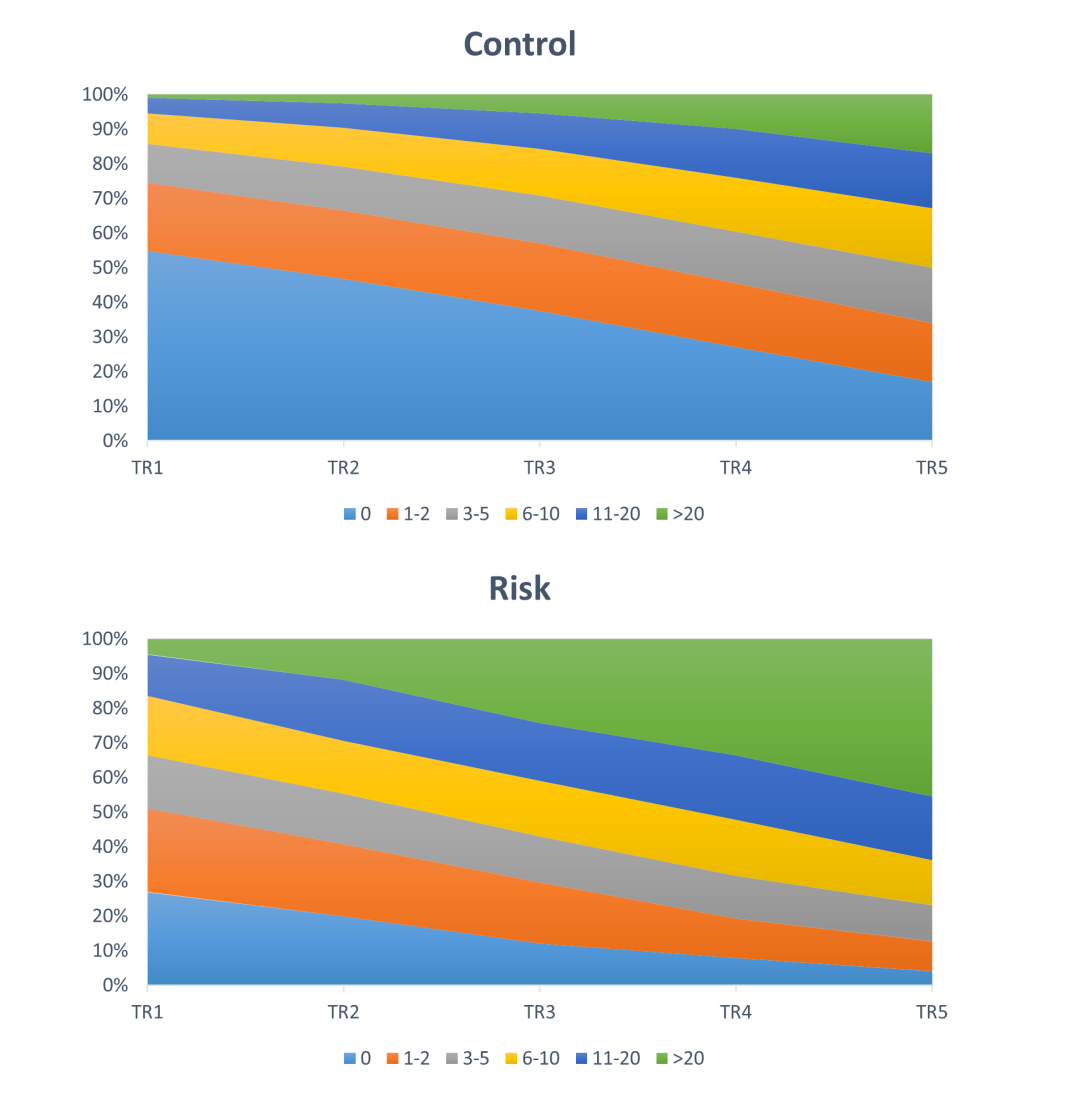
Distribution of assessments with respect to frequencies of ICD-codes (without chapter V-Mental and behavioral disorders) and time periods. Different color represents the frequencies of ICD-codes.

**Figure 8:**
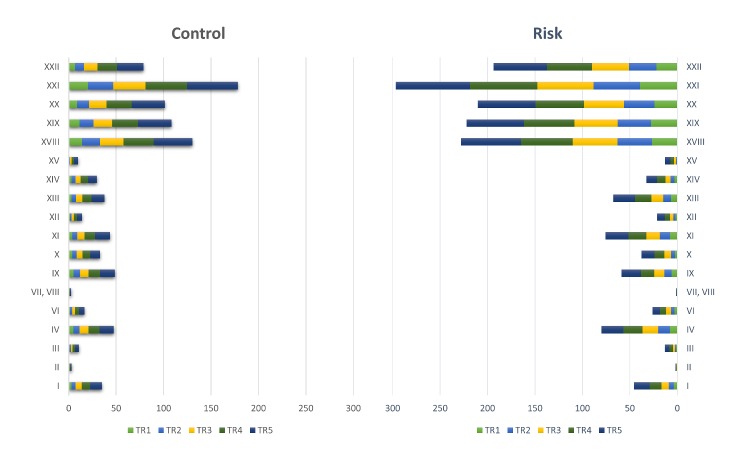
Cumulative percentage of cases in control and suicide risk groups over five time-periods grouped on ICD-10 category (Excluded chapters: chapter V-Mental and behavioral disorders, chapter XVI-Certain conditions originating in the perinatal period, and chapter XVII-Congenital malformations, deformations and chromosomal abnormalities).

Multiple ICD codes were more common among risk cases relative to the control cases (Table 1, Figure 7). For *τ=1* (0-3 months) percentages of risk cases were always higher than control cases for ICD codes more than zero. This trend changed with the increasing time, length where distribution of control populations become approximately equal over all five frequency ranges used in this study (Figure 7, top panel). On the other hand, for risk population the frequency of ICD codes increased with time ranges and therefore, approximately 45.46% (942/2072) of the total population had ICD code frequency >20 (Figure 8, bottom panel).

The prevalence of physical illness of both suicide risk and control groups grouped according to ICD-10 categories (chapter headings) has been summarized in Table 2 and Figure 8. Except for ICD-10 chapters II (Neoplasms) and (VII, VIII) (Sensory organ disease), a significantly higher prevalence of physical illness was observed in suicide risk cases than in control cases. However, the percentages of populations in those two chapters were insignificant across all organs or systems of the body. Interestingly, the most prevalent physical illness found for both control and suicide risk groups across all time ranges was Factors influencing health status and contact with health services (ICD-10 Chapter XXI), where the percentage of population continually increased from 20.38% (3013/14,786) to 53.54% (7916/14,786) and 39.29% (814/2072) to 78.38% (1624/2072) for control and suicide risk groups, respectively (Table 2). Interestingly, a significant (454/2072, 21.91%) population showed prevalence of multiple ICD-10 chapters in suicide risk cases at shorter time ranges than control cases. For *τ=1* and *τ=2*, ICD-10 chapter XXI were prevalent in more than 20.00% (2957/14,786) of control cases in contrast with five chapters (XVIII, XIX, XX, XXI, XXII) in suicide risk cases. This indicates that comorbidity is more prevalent and observable in shorter time range in suicide risk cases than control cases (Figure 8).

The performance of proposed physical illnesses (without ICD-10 chapter V)-based suicide risk scoring model has been shown in Table 3. The AUC value using RiskScore was 0.64, 0.67, 0.68, 0.68, and 0.69 for individual time ranges. This sequential increment of ROC area values with increased length of history of physical illnesses shows longer history length provides better suicide risk assessment than the shorter one. The maximum AUC 0.71 was obtained using physical illnesses from all time ranges, which indicates that overlapping history of physical illnesses improves the performance of the model than using a physical illnesses from a single time-period. In addition, for all of the lengths of the history of physical illnesses the *RiskScore_Algorithm_* performed better than clinically assessed risk score *RiskScore_Clinical_* (AUC=0.56).

The performance of regression model output *RiskScore_combined_* has been shown in Table 3. Similar to physical illnesses based *RiskScore_Algorithm_*, the AUC values increased with increasing length of history of physical illnesses (AUC=0.65, 0.67, 0.68, 0.69, and 0.70, respectively) and maximum AUC=0.72 was obtained for history of physical illnesses of all time ranges. Although AUC values of multilinear regression model was higher than physical illnesses based model, the improvement is marginal and statistically insignificant.

**Table 2:** Distribution of suicide risk and control cases associated with diagnostic groups according to ICD-10 category for five different time ranges.

*τ=1* (0-3M)				*τ=2* (0-6M)				*τ=3* (0-12M)				*τ=4* (0-24M)				*τ=5* (0-48M)			
Control		Risk		Control		Risk		Control		Risk		Control		Risk		Control		Risk	
n	%	n	%	n	%	n	%	n	%	n	%	n	%	n	%	n	%	n	%
**I Certain infectious and parasitic diseases**																			
440	2.98	73	3.52	638	4.31	115	5.55	944	6.38	157	7.58	1352	9.14	259	12.5	1816	12.28	346	16.7
**II Neoplasms**																			
28	0.19	1	0.05	45	0.30	5	0.24	67	0.45	7	0.34	131	0.89	10	0.48	194	1.31	17	0.82
**III Diseases of the blood and blood-forming organs and certain disorders involving the immune mechanism**																			
142	0.96	22	1.06	190	1.28	25	1.21	269	1.82	47	2.27	417	2.82	78	3.76	577	3.90	100	4.83
**IV Endocrine, nutritional and metabolic diseases**																			
724	4.90	165	7.96	994	6.72	252	12.16	1331	9.00	340	16.41	1740	11.77	420	20.27	2223	15.03	483	23.31
**VI Diseases of the nervous system**																			
230	1.56	60	2.90	324	2.19	79	3.81	454	3.07	104	5.02	599	4.05	134	6.47	858	5.80	166	8.01
**VII, VIII Sensory organ disease**																			
16	0.11	1	0.05	26	0.18	1	0.05	46	0.31	3	0.14	102	0.69	9	0.43	184	1.24	9	0.43
**IX Diseases of the circulatory system**																			
748	5.06	120	5.79	1022	6.91	163	7.87	1347	9.11	220	10.62	1766	11.94	291	14.04	2329	15.75	426	20.56
**X Diseases of the respiratory system**																			
548	3.71	53	2.56	700	4.73	88	4.25	898	6.07	142	6.85	1199	8.11	216	10.42	1533	10.37	282	13.61
**XI Diseases of the digestive system**																			
534	3.61	154	7.43	790	5.34	227	10.96	1145	7.74	297	14.33	1650	11.16	384	18.53	2347	15.87	502	24.23
**XII Diseases of the skin and subcutaneous tissue**																			
188	1.27	37	1.79	257	1.74	51	2.46	345	2.33	71	3.43	521	3.52	113	5.45	755	5.11	166	8.01
**XIII Diseases of the musculoskeletal system and connective tissue**																			
428	2.89	138	6.66	699	4.73	171	8.25	1004	6.79	256	12.36	1412	9.55	361	17.42	2037	13.78	471	22.73
**XIV Diseases of the genitourinary system**																			
444	3.00	57	2.75	598	4.04	86	4.15	844	5.71	113	5.45	1098	7.43	183	8.83	1389	9.39	237	11.44
**XV Pregnancy, childbirth and the puerperium**																			
90	0.61	8	0.39	152	1.03	15	0.72	216	1.46	45	2.17	386	2.61	85	4.10	617	4.17	114	5.50
**XVIII Symptoms, signs and abnormal clinical and laboratory findings, not elsewhere classified**																			
2079	14.06	547	26.40	2804	18.96	759	36.63	3660	24.75	978	47.20	4690	31.72	1125	54.3	6041	40.86	1311	63.27
**XIX Injury, poisoning and certain other consequences of external causes**																			
1677	11.34	574	27.70	2175	14.71	729	35.18	2934	19.84	946	45.66	4023	27.21	1095	52.85	5205	35.20	1254	60.52
**XX External causes of morbidity and mortality**																			
1315	8.89	495	23.89	1867	12.63	668	32.24	2727	18.44	872	42.08	3912	26.46	1064	51.35	5169	34.96	1260	60.81
**XXI Factors influencing health status and contact with health services**																			
3013	20.38	814	39.29	3911	26.45	1010	48.75	5053	34.17	1231	59.41	6476	43.80	1470	70.95	7916	53.54	1624	78.38
**XXII Codes for special purposes**																			
989	6.69	455	21.96	1391	9.41	595	28.72	2086	14.11	808	39.00	3070	20.76	984	47.49	4106	27.77	1165	56.23

**Table 3:** AUC obtained using suicide risk scoring model based on physical illnesses, clinically assessed score and their combinations.

Length of history of physical illness	AUC		
	*RiskScore_Algorithm_*	*RiskScore_Clinical_*	*RiskScore_Combined_*
0-3 months (τ=1)	0.64	0.56	0.65
0-6 months (τ=2)	0.67	0.56	0.67
0-12 months (τ=3)	0.68	0.56	0.68
0-24 months (τ=4)	0.68	0.56	0.69
0-48 months (τ=5)	0.69	0.56	0.70
Combined τ (1, 2, … 5)	0.71	0.56	0.72

## Discussion

### Principal Findings

To our knowledge, this study is the first to only use the patient’s history of physical illnesses (ICD-10 codes, without chapter V, Mental and behavioral disorders) to predict suicide risk. This study has demonstrated how to exploit the physical illnesses to predict suicide risk using EMR of a single regional hospital (Barwon Health). The ready availability of EMR shows promise that such tools can be integrated within hospital systems for effective decision support.

The findings of this study, based on all patients of Barwon Health who had the mandated suicide risk assessment between April 2009 and March 2012, showed that the percentage of the population having a history of physical illness were higher in risk group than the control. This supports previously reported findings that hospitalization for a physical illness significantly increases the risk of subsequent suicide [8,28]. Although this higher prevalence of physical illnesses in our risk group was found over five different time-periods ranging from 3 to 48 months, the difference in prevalence between two groups decreased with increasing time (Table 1, Figure 8). This indicates that time-period over which history is considered is a critical parameter in predicting risk.

The results of this study showed that the frequency of physical illness (11-20, >20) was higher in suicide risk population than control for all time periods, which is similar to the findings reported by Qin et al [28]. However, for smaller frequency values, the percentage of control cases exceeded the percentage of suicide risk cases with increasing historical time-period. Although this findings are different from Qin et al [28] where they have used much longer time period than 48 months, this can be attributed to the cohort difference-they have reported 1.13% of suicide cases with physical illnesses frequency >20 in contrast to 0.27% of control; in our study for a 48-month period, we found these frequencies to be 45.46% (942/2072) and 17.04% (2519/14,786) for the risk and control group, respectively.

The performance of ICD-10 based (without chapter V) suicide risk score, *RiskScore_Algorithm_* performed better than 18-point risk checklist based clinical assessment (*RiskScore_Clinical_*) for all time-periods used in this study. This indicates that 3 or more months of history of physical illnesses can better predict the suicide risk than clinical assessment. This supports the previous findings that additional information is required in designing a more effective and automated suicide risk assessment systems suitable for clinical settings [13,14]. *RiskScore_Combined_* showed a marginal improvement in suicide risk prediction than physical illnesses based score *RiskScore_Algorithm_* but substantial improvement over clinical assessment. Therefore, adding history of physical illnesses with regular clinical assessments can improve the performance of suicide risk prediction. Since physical illnesses based models were tested using 10-fold cross validation, the performance can be considered to be robust.

A limitation of this study is that we have considered only physical illnesses that resulted in hospitalization. However, this is an inherent and unavoidable limitation of any study based on hospital records. Therefore, the effect of mild illnesses that resulted in no hospitalization or treated outside hospitals was not considered. Furthermore, we did not consider the effect of age or gender on the distribution of physical illnesses and developed a single model for scoring the suicide risk, which may provide some bias. The small number of suicide risk cases restricted us from stratifying by age or gender as this would result in a sparse lookup table.

This study has following clinical implications: (1) The results of this study shows that hospital clinicians who are not specialists in mental health can use our decision support tool for identifying patients at risk of attempt of suicide and this may improve patient care, (2) clinical assessors with mental health expertise can use patient’s history of physical illnesses through our proposed tool to improve the prediction of risk of suicide attempt, especially for patients with a history of multiple hospitalizations, and (3) our tool can also assist primary care providers with access to EMR to recognize early signs of risk of suicide attempt and refer patients to specialty care.

### Conclusion

In summary, this study provides a novel approach to exploit the history of physical illnesses extracted from EMR (ICD-10 codes without chapter V-Mental and behavioural disorders) to predict risk of suicide attempt. This model also outperforms existing clinical assessments of suicide risk.

## Abbreviations

AUC: area under the receiver operating characteristic curve
EMR: electronic medical records
ICD-10: International Statistical Classification of Diseases and Related Health Problems, 10th Revision
